# Cardiac defibrillator implantation in patients with syncope and inducible ventricular arrhythmia: insights from the German Device Registry

**DOI:** 10.1038/s41598-023-37440-2

**Published:** 2023-07-27

**Authors:** Ann-Kathrin Kahle, Jochen Senges, Matthias Hochadel, Johannes Brachmann, Dierk Thomas, Florian Straube, Klaus Bonaventura, Robert Larbig, Nikos Werner, Christian Butter, Fares-Alexander Alken, Christian Meyer

**Affiliations:** 1 Division of Cardiology, Angiology, Intensive Care Medicine, EVK Düsseldorf, cNEP, Cardiac Neuro- and Electrophysiology Research Consortium, Kirchfeldstrasse 40, 40217 Düsseldorf, Germany; 2 Department of Cardiology, Pulmonology and Vascular Medicine, Medical Faculty, University Hospital Düsseldorf, Düsseldorf, Germany; 3 Institute of Neural and Sensory Physiology, Medical Faculty, cNEP, Cardiac Neuro- and Electrophysiology Research Consortium, Heinrich Heine University Düsseldorf, Düsseldorf, Germany; 4 Stiftung Institut für Herzinfarktforschung, Ludwigshafen, Germany; 5 Medical School REGIOMED, Coburg, Germany; 6 University of Split School of Medicine, Split, Croatia; 7 Department of Cardiology, Medical University Hospital, Heidelberg, Germany; 8 DZHK (German Centre for Cardiovascular Research), Partner Site Heidelberg/Mannheim, Heidelberg, Germany; 9 Department of Cardiology and Internal Intensive Care Medicine, Heart Center Munich-Bogenhausen, Munich, Germany; 10 Department of Internal Medicine/Cardiology and Angiology, Ernst-Von-Bergmann Clinic, Potsdam, Germany; 11 Division of Cardiology, Hospital Maria Hilf Mönchengladbach, Mönchengladbach, Germany; 12 Department of Cardiology, University Heart Centre Bonn, Bonn, Germany; 13 Medical Department III, Krankenhaus der Barmherzigen Brüder Trier, Trier, Germany; 14 Department of Cardiology, Heart Center Brandenburg Bernau, Bernau, Germany

**Keywords:** Cardiac device therapy, Neuro-vascular interactions

## Abstract

History of syncope is an independent predictor for sudden cardiac death. Programmed stimulation may be considered for risk stratification, but data remain sparse among different populations. Here, we analyzed the prognostic value of inducible ventricular arrhythmia (VA) regarding clinical outcome in patients with syncope undergoing defibrillator implantation. Among 4196 patients enrolled in the prospective, multi-center German Device Registry, patients with syncope and inducible VA (n = 285, 6.8%) vs. those with a secondary preventive indication (n = 1885, 45.2%), defined as previously documented sustained ventricular tachycardia or ventricular fibrillation, serving as a control group were studied regarding demographics, device implantation and post-procedural adverse events. Patients with syncope and inducible VA (64.9 ± 14.4 years, 81.1% male) presented less frequently with congestive heart failure (15.1% vs. 29.1%; *p* < 0.001) and any structural heart disease (84.9% vs. 89.3%; *p* = 0.030) than patients with a secondary preventive indication (65.0 ± 13.8 years, 81.0% male). Whereas dilated cardiomyopathy (16.8% vs. 23.8%; *p* = 0.009) was less common, hypertrophic cardiomyopathy (5.6% vs. 2.8%; *p* = 0.010) and Brugada syndrome (2.1% vs. 0.3%; *p* < 0.001) were present more often. During 1-year-follow-up, mortality (5.1% vs. 8.9%; *p* = 0.036) and the rate of major adverse cardiac or cerebrovascular events (5.8% vs. 10.0%; *p* = 0.027) were lower in patients with syncope and inducible VA. Among patients with inducible VA, post-procedural adverse events including rehospitalization (27.6% vs. 21.7%; *p* = 0.37) did not differ between those with vs. without syncope. Taken together, patients with syncope and inducible VA have better clinical outcomes than patients with a secondary preventive defibrillator indication, but comparable outcomes to patients without syncope, which underlines the relevance of VA inducibility, potentially irrespective of a syncope.

## Introduction

The implantable cardioverter-defibrillator (ICD) is the mainstay of therapy for the primary and secondary prevention of sudden cardiac death (SCD)^1^. Programmed ventricular stimulation (PVS) is indicated for risk stratification of SCD in post-infarct patients in whom syncope remains unexplained after non-invasive evaluation. It may further be considered for selected populations including patients with dilated or arrhythmogenic right ventricular cardiomyopathy^2^. In this context, it has been confirmed that patients with coronary artery disease, left ventricular dysfunction and inducible sustained ventricular arrhythmia (VA) during PVS have a higher risk of SCD and a higher overall mortality than those without inducible VA^3^. For other patient populations data remain controversial.

The history of syncope is an independent predictor for SCD as well as appropriate device discharge and may be helpful for identification of patients at increased risk^4^. However, patients with syncope and inducible VA during PVS, comprising a small group of all patients undergoing defibrillator implantation, are not well represented in clinical trials and data on the prognostic value of inducible VA regarding clinical outcome during the first time period after ICD implantation in patients with syncope are sparse^5^. Therefore, the purpose of this study was to assess patient characteristics, data on device implantation and post-procedural adverse events in patients with syncope and inducible VA, derived from a prospective, multi-center database, compared to those with a secondary preventive indication, defined as previously documented occurrence of either sustained ventricular tachycardia or ventricular fibrillation, serving as a well-studied control group with respect to ICD implantation as a gold standard for reducing the rate of mortality and major adverse cardiac or cerebrovascular events (MACCE). Considering the limited indications for PVS, in an additional analysis of all patients with inducible VA, those with vs. without previous syncope were compared for patient characteristics and clinical outcomes after defibrillator implantation.

## Methods

### Recruitment and study design

The German Device Registry is a prospective, multi-center non-profit database of ICD implantations with or without cardiac resynchronization therapy (CRT) under supervision of the “Stiftung Institut für Herzinfarktforschung” (IHF, Ludwigshafen, Germany). A total of 50 participating centers enrolled patients between March 2007 and April 2010. The ethics committee of the Rhineland‐Palatinate State Medical Council (Landesärztekammer Rheinland-Pfalz) approved the study, which was conducted in accordance with the provisions of the Declaration of Helsinki, all relevant guidelines and regulations. Patients gave written informed consent for procedure and registry participation. All data were entered into an internet-based, secure electronic database and transmitted to the IHF using a secure socket layer for encryption. The registry captured baseline data on patients’ demographics, device indication, implantation procedure and complications^6^.

The present study population includes patients undergoing ICD implantation or replacement divided into patients with a history of at least one syncope and inducible VA, defined as inducible sustained monomorphic ventricular tachycardia or ventricular fibrillation, during PVS vs. patients with a secondary preventive indication, defined as previously documented occurrence of either sustained ventricular tachycardia or ventricular fibrillation, serving as a well-studied control group. PVS was performed in line with the given ESC guidelines as interpreted at the operator’s discretion. In an additional analysis of patients with inducible VA, those with vs. without previous syncope were compared.

### Follow-up

Follow-up contacts were scheduled 1 year after device implantation or replacement by telephone and performed by IHF representatives. The telephone interview included questions on arrhythmias, cardiac events, clinical symptoms, medication, complications, hospitalizations and quality of life. In case of an ineffective call, further information was gathered from other caring physicians or civil registration offices. Data on mortality and ICD shocks were additionally obtained from available records and device interrogations^6^.

### Systematic literature review

To put our presented findings from a large prospective, multi-center database into context, we performed a systematic literature review on prospective studies assessing patients with previous syncope and inducible VA during PVS. The literature search was conducted using the terms “programmed ventricular stimulation AND syncope” OR “inducible ventricular tachycardia AND syncope” OR “ventricular stimulation AND syncope” OR “VT induction AND syncope” in PubMed/MEDLINE. We supplemented our database searches with manual searches of the reference lists of published studies. Potentially eligible studies were assessed independently by 2 investigators (A.K.K. and C.M.). Studies were included if they (1) assessed patients with syncope and inducible VA; and (2) reported demographics and/or data on ICD implantation and/or outcomes (after ICD implantation) in this population. The following exclusion criteria were applied: (1) Case reports and case series; (2) studies published in abstract form only; (3) studies of which the abstract was not available in English; (4) studies investigating arrhythmias other than inducible VA; (5) studies only including patients with documented ion channel diseases. The initial review included all prospective and retrospective studies with or without a control group and was subsequently restricted to prospective studies only.

In addition, we reviewed classes of recommendations and levels of evidence for ICD implantation in patients with syncope and inducible VA according to international guidelines^2,7,8^.

### Statistical analysis

Continuous variables are presented as mean ± SD or median with interquartile ranges (25th–75th percentile). Categorical variables are expressed as absolute numbers with percentage of patients. Statistical differences between groups were compared using the Mann–Whitney *U* test, the chi-square test or the Fisher’s exact test, respectively. Survival analysis was estimated using the Kaplan–Meier method and compared with the log-rank test. A *p* value < 0.05 was considered statistically significant. All analyses were performed at the Biometrics Department of the IHF.

## Results

### Study population

**Figure 1:**
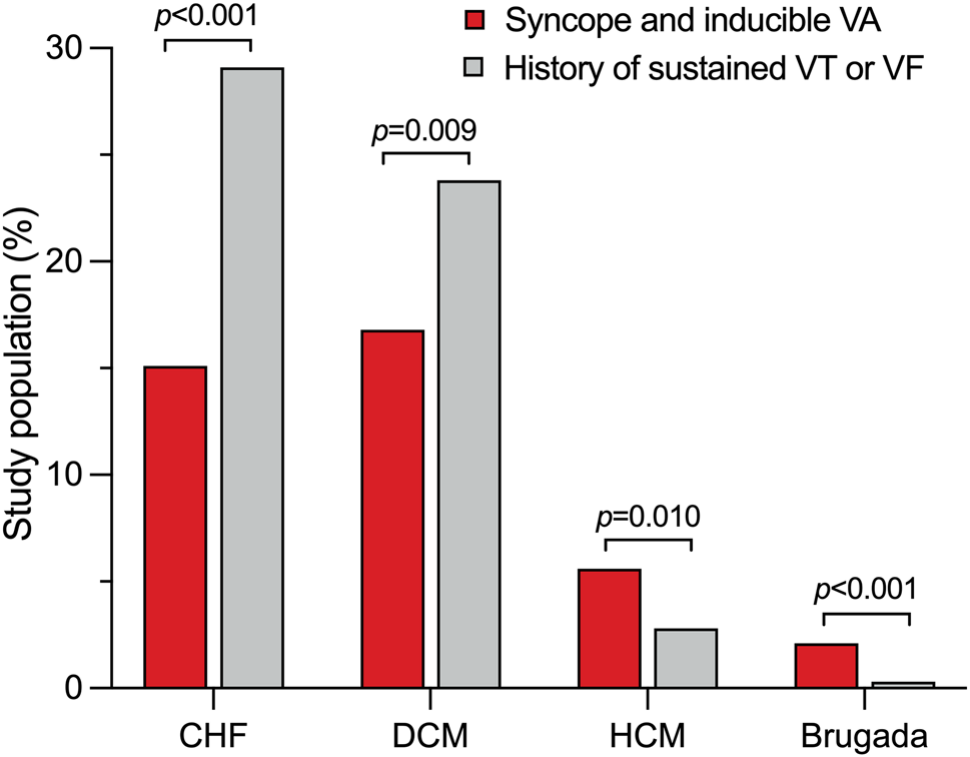
Cardiac comorbidities of the study population. The incidence of cardiac comorbidities in patients with syncope and inducible VA versus patients with a history of sustained VT or VF is shown. Whereas CHF and DCM were less common in patients with syncope and inducible VA, HCM and Brugada syndrome were found more frequently than in patients with a history of sustained VT or VF. *CHF* congestive heart failure, *DCM* dilated cardiomyopathy, *HCM* hypertrophic cardiomyopathy, *VA* ventricular arrhythmia, *VF* ventricular fibrillation, *VT* ventricular tachycardia.

Out of 4196 patients enrolled in the German Device Registry, 285 (6.8%) had a history of syncope or presyncope and inducible VA during PVS, 1885 patients (45.2%) had a secondary preventive defibrillator indication with documented ventricular tachycardia in 51.8% and ventricular fibrillation in 45.8% of patients. Demographic data at baseline are shown in Table 1. Patients with syncope and inducible VA presented less often with congestive heart failure, defined as the presence of diagnostic signs and symptoms of heart failure according to the ESC guidelines, (15.1% vs. 29.1%; *p* < 0.001) and any underlying structural heart disease (84.9% vs. 89.3%; *p* = 0.030) than patients with a secondary preventive indication. Whereas dilated cardiomyopathy (16.8% vs. 23.8%; *p* = 0.009) was less common in patients with syncope and inducible VA, hypertrophic cardiomyopathy (5.6% vs. 2.8%; *p* = 0.010) and Brugada syndrome (2.1% vs. 0.3%; *p* < 0.001) were present more frequently (Fig. 1). They had a higher left ventricular ejection fraction (43.7 ± 14.2% vs. 38.1 ± 14.2%; *p* < 0.001) with a lower percentage of patients assigned to left ventricular ejection fraction ≤ 30% (27.8% vs. 42.7%; *p* < 0.001) and New York Heart Association class III–IV (21.7% vs. 30.0%; *p* = 0.008). Intraventricular conduction disorders were found less frequently in patients with previous syncope and inducible VA (30.2% vs. 36.6%; *p* = 0.035). Previous cardiopulmonary resuscitation was reported in 55.4% of patients undergoing secondary preventive defibrillator implantation. The rate of previous sudden cardiac death in the family history did not differ between groups (3.6% vs. 4.4%; *p* = 0.84). Demographic data of patients undergoing first-time defibrillator implantation are shown in Supplementary Table S1.

**Table 1:** Patient baseline characteristics.

Variable	Syncope and inducible VA (n = 285)	Secondary preventive indication (n = 1885)	*p* value	Odds ratio (95%-CI)
Age, years	64.9 ± 14.4	65.0 ± 13.8	0.93	–
Male sex	231 (81.1)	1526 (81.0)	0.97	1.01 (0.73–1.38)
Body mass index, kg/m^2^	25.0 (23.5–29.7)	26.1 (24.2–29.4)	0.60	–
LVEF, %	43.7 ± 14.2	38.1 ± 14.2	< 0.001	–
LVEF ≤ 30%	73 (27.8)	758 (42.7)	< 0.001	0.51 (0.39–0.69)
Sinus rhythm at baseline	231 (81.1)	1459 (77.6)	0.19	1.23 (0.90–1.69)
Atrial fibrillation at baseline	41 (14.4)	338 (18.0)	0.14	0.77 (0.54–1.09)
Atrioventricular block	57 (20.0)	346 (18.4)	0.53	1.11 (0.81–1.51)
Atrioventricular block III	0 (0)	23 (6.8)	0.16	–
Intraventricular conduction disorder	86 (30.2)	687 (36.6)	0.035	0.75 (0.57–0.98)
LBBB	51 (17.9)	398 (21.2)	0.20	0.81 (0.59–1.12)
RBBB	12 (4.2)	160 (8.5)	0.012	0.47 (0.26–0.86)
Structural heart disease	242 (84.9)	1683 (89.3)	0.030	0.68 (0.47–0.96)
Coronary artery disease	162 (56.8)	1172 (62.2)	0.085	0.80 (0.62–1.03)
Prior myocardial infarction	95 (33.3)	683 (36.2)	0.34	0.88 (0.68–1.15)
Time since myocardial infarction, months	143 (111–226)	78 (2–168)	0.002	–
Coronary artery bypass graft	38 (13.3)	325 (17.2)	0.099	0.74 (0.51–1.06)
Dilated cardiomyopathy	48 (16.8)	448 (23.8)	0.009	0.65 (0.47–0.90)
Hypertrophic cardiomyopathy	16 (5.6)	52 (2.8)	0.010	2.10 (1.18–3.72)
Congestive heart failure	43 (15.1)	548 (29.1)	< 0.001	0.43 (0.31–0.61)
Time since diagnosis, months	4 (0–30)	3 (0–20)	1	–
Hypertensive heart disease	18 (6.3)	86 (4.6)	0.20	1.41 (0.83–2.38)
Primary electrical disease	15 (5.3)	71 (3.8)	0.23	1.42 (0.80–2.51)
Brugada syndrome	6 (2.1)	6 (0.3)	< 0.001	6.73 (2.16–21.02)
Long QT	2 (0.7)	38 (2.0)	0.12	0.34 (0.08–1.43)
ARVC	3 (1.1)	16 (0.8)	0.73	1.24 (0.36–4.29)
Arterial hypertension	149 (52.3)	905 (48.0)	0.18	1.19 (0.92–1.52)
Diabetes mellitus	48 (16.8)	421 (22.3)	0.036	0.70 (0.51–0.98)
Stroke	7 (2.5)	77 (4.1)	0.18	0.59 (0.27–1.29)
Chronic obstructive pulmonary disease	2 (0.7)	51 (2.7)	0.041	0.25 (0.06–1.05)
Chronic kidney disease	29 (10.2)	265 (14.1)	0.074	0.69 (0.46–1.04)

Values are presented as mean ± SD, median (IQR) or n (%). Variables were compared using the Mann–Whitney *U* test or the chi-square test, respectively.

*ARVC* arrhythmogenic right ventricular cardiomyopathy, *CI* confidence interval, *LBBB* left bundle branch block, *LVEF* left ventricular ejection fraction, *RBBB* right bundle branch block, *VA* ventricular arrhythmia.

### Implantation procedure

Device implantation was performed more often urgently in patients with syncope and inducible VA than in patients with a secondary preventive indication (46.4% vs. 22.5%; *p* = 0.005). Of all procedures, 77.5% were first-time implants, with fewer previous implants in the cohort of patients with syncope and inducible VA (31.5% vs. 40.0%; *p* = 0.010). Previous implants were mainly ICDs without (27.2% vs. 34.6%; *p* = 0.020) or with CRT (0.4% vs. 2.8%; *p* = 0.021). Pacemakers were implanted in 4.3% and 4.8% of patients, respectively (*p* = 0.71).

During the index implantation procedure, single-chamber ICDs were chosen less frequently in patients with a history of syncope and inducible VA during PVS (48.8% vs. 60.1%; *p* < 0.001), and dual-chamber ICDs more often than in patients with a secondary preventive indication (40.7% vs. 26.0%; *p* < 0.001). ICDs with CRT were implanted in 10.5% vs. 13.9%, respectively (*p* = 0.12).

Rates of revision of the implanted or replaced defibrillator (1.7% vs. 2.3%; *p* = 0.64) as well as complications (2.4% vs. 3.5%; *p* = 0.45) during hospital stay did not differ between groups (Table 2). At discharge, patients with syncope and inducible VA were treated less often with class III antiarrhythmic drugs (10.6% vs. 21.7%; *p* < 0.001) and digitalis (9.6% vs. 14.4%; *p* = 0.029) (Table 3).

**Table 2:** Peri-procedural and in-hospital complications.

Variable	Syncope and inducible VA (n = 285)	Secondary preventive indication (n = 1885)	*p* value	Odds ratio (95%-CI)
Death	1 (0.4)	11 (0.6)	0.62	0.60 (0.08–4.66)
For cardiac reasons	1 (100.0)	5 (45.5)	0.30	–
Survival time, days	14	12 (5–22)	1	–
MACCE	1 (0.4)	11 (0.7)	1	0.54 (0.07–4.23)
Complication requiring intervention				
Pericardial effusion	0 (0)	3 (0.2)	1	–
Hemothorax	0 (0)	3 (0.2)	1	–
Pneumothorax	0 (0)	9 (0.5)	0.62	–
Pocket hematoma	2 (0.7)	22 (1.2)	0.76	0.60 (0.14–2.56)
Complication not requiring intervention				
Pericardial effusion	1 (0.4)	1 (0.1)	0.27	6.01 (0.37–96.42)
Hemothorax	0 (0)	1 (0.1)	1	–
Pneumothorax	1 (0.4)	2 (0.1)	0.37	3.01 (0.27–33.27)
Pocket hematoma	1 (0.4)	7 (0.4)	1	0.94 (0.12–7.70)
Other	2 (0.8)	19 (1.2)	0.76	0.63 (0.15–2.71)

Values are presented as n (%) or median (IQR). Variables were compared using the Fisher’s exact test or the Mann–Whitney *U* test.

*CI* confidence interval, *MACCE* major adverse cardiac or cerebrovascular event, *VA* ventricular arrhythmia.

**Table 3:** Medication of the study population.

Variable	At discharge				At 1-year-follow-up			
	Syncope and inducible VA (n = 285)	Secondary preventive indication (n = 1885)	*p* value	Odds ratio (95%-CI)	Syncope and inducible VA (n = 285)	Secondary preventive indication (n = 1885)	*p* value	Odds ratio (95%-CI)
Beta-receptor blocker	88.3	87.8	0.80	1.05 (0.71–1.55)	86.4	84.3	0.42	1.19 (0.78–1.81)
Class I antiarrhythmic drug	2.1	1.5	0.47	1.39 (0.57–3.37)	3.7	1.8	0.071	2.09 (0.92–4.73)
Class III antiarrhythmic drug	10.6	21.7	< 0.001	0.43 (0.29–0.64)	18.7	21.6	0.34	0.84 (0.58–1.21)
Amiodarone	93.3	94.8	0.72	0.76 (0.17–3.41)	92.5	91.9	0.90	1.09 (0.31–3.81)
Sotalol	0	5.4	0.19	–	2.5	7.4	0.25	0.32 (0.05–2.48)
Dronedarone	0	0	–	–	5.0	0.7	0.025	7.11 (0.97–51.94)
Digitalis	9.6	14.4	0.029	0.63 (0.42–0.96)	8.4	13.9	0.027	0.57 (0.34–0.94)
Anticoagulation	14.3	32.9	0.041	0.34 (0.11–1.00)	33.3	34.4	0.92	0.95 (0.39–2.33)
Platelet aggregation inhibitor	57.1	57.9	0.94	0.97 (0.45–2.11)	50.0	47.7	0.83	1.10 (0.47–2.55)
ACE inhibitor/AT1 receptor antagonist	76.6	81.8	0.039	0.73 (0.54–0.99)	71.5	75.8	0.18	0.80 (0.58–1.11)
Aldosterone receptor antagonist	16.7	31.7	< 0.001	0.43 (0.31–0.60)	19.6	28.4	0.008	0.62 (0.43–0.88)
Diuretics	52.1	61.9	0.002	0.67 (0.52–0.86)	46.3	58.4	< 0.001	0.61 (0.46–0.82)
Statin	60.3	59.3	0.76	1.04 (0.81–1.34)	56.5	56.7	0.98	1.00 (0.74–1.33)

Values are presented as %. Variables were compared using the chi-square test.

*CI* confidence interval, *VA* ventricular arrhythmia.

### Follow-up

**Figure 2:**
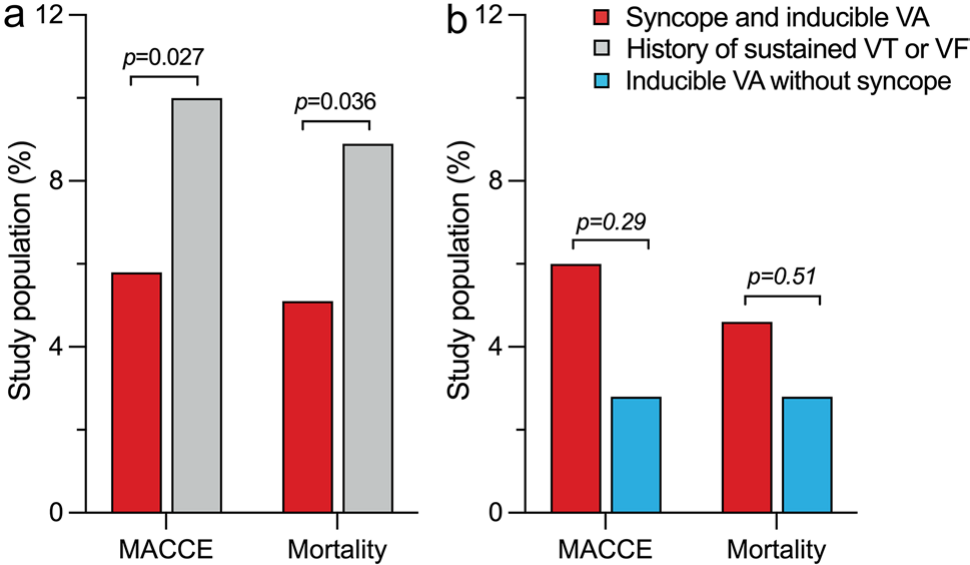
Main endpoints during 1-year-follow-up. Main endpoints after device implantation or revision are shown for patients with syncope and inducible VA versus (**a**) patients with a history of sustained VT or VF and (**b**) patients with inducible VA without syncope. Patients with previous syncope and inducible VA had lower rates of 1-year-mortality and MACCE than patients with a history of sustained VT or VF, but similar rates compared to patients undergoing defibrillator implantation for inducible VA without previous syncope. *MACCE* major adverse cardiac or cerebrovascular event, *VA* ventricular arrhythmia, *VF* ventricular fibrillation, *VT* ventricular tachycardia.

During a median follow-up of 16.4 [IQR 12.8–24.3] months vs. 17.5 [IQR 13.3–25.0] months (*p* = 0.022), 1-year-mortality (5.1% vs. 8.9%; *p* = 0.036) and the rate of MACCE (5.8% vs. 10.0%; *p* = 0.027) were lower in patients with syncope and inducible VA compared to patients with a secondary preventive indication (Fig. 2a). Other events did not differ (Table 4). At 1-year-follow-up, digitalis (8.4% vs. 13.9%; *p* = 0.027), aldosterone antagonists (19.6% vs. 28.4%; *p* = 0.008) and diuretics (46.3% vs. 58.4%; *p* < 0.001) were applied less often in patients with a history of syncope and inducible VA, dronedarone (5.0% vs. 0.7%; *p* = 0.025), but not class III antiarrhythmic drugs in general (18.7% vs. 21.6%; *p* = 0.34), was applied more often (Table 3). Clinical outcomes during 1 year of follow-up of patients undergoing first-time defibrillator implantation are shown in Supplementary Table S2.

**Table 4:** Post-procedural adverse events during 1 year of follow-up.

Variable	Syncope and inducible VA (n = 285)	Secondary preventive indication (n = 1,885)	*p* value	Odds/hazard ratio (95%-CI)
1-year-mortality^a^	5.1	8.9	0.036	0.56 (0.33–0.97)
Survival time, months	11.0 (4.1–19.0)	16.6 (13.0–22.3)	0.86	–
MACCE	5.8	10.0	0.027	0.56 (0.33–0.94)
Cardiopulmonary resuscitation	0	0.9	0.15	–
Syncope	4.8	6.1	0.53	0.77 (0.34–1.73)
ICD shock	18.6	23.0	0.14	0.76 (0.53–1.09)
VT storm / incessant VT	2.4	2.3	0.71	1.18 (0.49–2.87)
Ablation procedure	7.4	5.2	0.33	1.46 (0.68–3.14)
Myocardial infarction	1.4	1.1	0.67	1.31 (0.37–4.60)
Stroke	0	1.2	0.11	–
Revascularization	3.1	3.3	0.90	0.93 (0.32–2.70)
Device revision	6.3	9.4	0.14	0.65 (0.37–1.15)
Rehospitalization	36.9	41.4	0.21	0.83 (0.61–1.11)
For device-related reasons	15.7	16.4	0.77	0.94 (0.64–1.40)
For other cardiac reasons	8.3	10.7	0.29	0.76 (0.45–1.27)
Number of hospitalizations	1 (1–2)	1 (1–2)	0.26	–
Duration of hospitalization, days	9 (4–21)	9 (5–19)	0.88	–

Values are presented as % or median (IQR). Variables were compared using the chi-square or the Mann–Whitney *U* test.

^a^Kaplan-Meier estimates at 366 days after index discharge, compared by log-rank test (hazard ratio).

*CI* confidence interval, *ICD* implantable cardioverter-defibrillator, *MACCE* major adverse cardiac or cerebrovascular event, *VA* ventricular arrhythmia, *VT* ventricular tachycardia.

### Patients with inducible VA

In an additional analysis of patients with inducible VA, defined as inducible sustained monomorphic ventricular tachycardia or ventricular fibrillation during PVS, 257 (6.2%) patients with a history of at least one syncope were compared to 86 (2.1%) without previous syncope, who underwent PVS mainly for diagnostic evaluation after remote myocardial infarction with symptoms such as palpitations suggestive of VA. Patients with syncope presented less often with coronary artery disease (56.4% vs. 70.9%; *p* = 0.017) and prior myocardial infarction (33.1% vs. 46.5%; *p* = 0.025) than those without syncope (Table 5).

**Table 5:** Baseline characteristics of patients with inducible VA.

Variable	Syncope and inducible VA (n = 257)	Inducible VA without syncope (n = 86)	*p* value	Odds ratio (95%-CI)
Age, years	65.1 ± 14.6	65.5 ± 12.2	0.72	–
Male sex	209 (81.3)	74 (86.0)	0.32	0.71 (0.36–1.40)
LVEF, %	43.7 ± 14.5	39.9 ± 15.4	0.059	–
Sinus rhythm at baseline	204 (79.4)	76 (88.4)	0.062	0.51 (0.25–1.05)
Atrial fibrillation at baseline	40 (15.6)	8 (9.3)	0.15	1.80 (0.81–4.01)
Atrioventricular block	54 (21.0)	17 (19.8)	0.81	1.08 (0.59–1.99)
Intraventricular conduction disorder	76 (29.6)	31 (36.0)	0.26	0.74 (0.44–1.25)
LBBB	47 (18.3)	15 (17.4)	0.86	1.06 (0.56–2.01)
RBBB	9 (3.5)	6 (7.0)	0.17	0.48 (0.17–1.40)
Coronary artery disease	145 (56.4)	61 (70.9)	0.017	0.53 (0.31–0.90)
Prior myocardial infarction	85 (33.1)	40 (46.5)	0.025	0.57 (0.35–0.93)
Coronary artery bypass graft	32 (12.5)	16 (18.6)	0.15	0.62 (0.32–1.20)
Cardiomyopathy	57 (22.2)	11 (12.8)	0.059	1.94 (0.97–3.90)
Dilated cardiomyopathy	41 (16.0)	10 (11.6)	0.33	1.44 (0.69–3.02)
Hypertrophic cardiomyopathy	16 (6.2)	1 (1.2)	0.062	5.64 (0.74–43.20)
Hypertensive heart disease	17 (6.6)	5 (5.8)	0.79	1.15 (0.41–3.21)
Primary electrical disease	14 (5.4)	3 (3.5)	0.47	1.59 (0.45–5.68)
Brugada syndrome	6 (2.3)	0 (0)	0.15	–
Long QT	2 (0.8)	0 (0)	0.41	–
ARVC	2 (0.8)	2 (2.3)	0.25	0.33 (0.05–2.37)
Arterial hypertension	138 (53.7)	46 (53.5)	0.97	1.01 (0.62–1.65)
Diabetes mellitus	46 (17.9)	16 (18.6)	0.88	0.95 (0.51–1.79)
Stroke	6 (2.3)	4 (4.7)	0.27	0.49 (0.13–1.78)
Chronic obstructive pulmonary disease	1 (0.4)	1 (1.2)	0.41	0.33 (0.02–5.37)
Chronic kidney disease	29 (11.3)	12 (14.0)	0.51	0.78 (0.38–1.61)

Values are presented as mean ± SD, median (IQR) or n (%). Variables were compared using the Mann–Whitney *U* test or the chi-square test, respectively.

*ARVC* arrhythmogenic right ventricular cardiomyopathy, *CI* confidence interval, *LBBB* left bundle branch block, *LVEF* left ventricular ejection fraction, *RBBB* right bundle branch block, *VA* ventricular arrhythmia.

Of all procedures, 76.4% were first-time implants, with a higher percentage in the cohort of patients with syncope (79.3% vs. 68.6%; *p* = 0.043). In patients who were included for device revision, the first implantation was conducted 5 years [IQR 4–7] before study enrollment in both groups (*p* = 0.44). Previous implants in patients with vs. without syncope were mainly ICDs without (27.2% vs. 33.7%; *p* = 0.25) or with CRT (0.4% vs. 3.5%; *p* = 0.020). Pacemakers were implanted in 4.3% and 3.5% of patients, respectively (*p* = 0.25).

The choice of the firstly implanted or revised device did not differ between groups, with an ICD without and with CRT implanted in 89.5% vs. 86.0% and 10.5% vs. 14.0% of patients (*p* = 0.38), respectively. Single-chamber ICDs were chosen in 55.7% vs. 48.8% (*p* = 0.51), dual-chamber ICDs in 44.7% vs. 37.2% (*p* = 0.22). Rates of revision of the implanted or replaced defibrillator (1.7% vs. 1.2%; *p* = 0.78) and complications (2.4% vs. 1.2%; *p* = 0.68) during hospital stay did not differ between groups.

During a median follow-up of 15.0 months [IQR 12.8–18.5] vs. 17.7 months [IQR 13.4–22.8] (*p* = 0.004), there were no differences between the cohorts in terms of 1-year-mortality (4.6% vs. 2.8%; *p* = 0.51), MACCE (6.0% vs. 2.8%; *p* = 0.29), recurrent syncope (3.0% vs. 0%; *p* = 0.32), ventricular tachycardia storm (1.7% vs. 0%; *p* = 0.31), ICD shocks (16.0% vs. 15.0%; *p* = 0.85), rehospitalization (27.6% vs. 21.7%; *p* = 0.37) and improvement in quality of life (44.0% vs. 47.5%; *p* = 0.64) (Fig. 2b), but the feeling of safety during treatment was higher among those with syncope (100.0% vs. 81.8%; *p* = 0.004). Demographic data and outcomes of patients with inducible VA with vs. without syncope undergoing first-time defibrillator implantation are shown in Supplementary Tables S3 and S4.

### Systematic literature review on patients with syncope and inducible VA

In addition to our here presented findings and to put them in relation to previously published reports, we performed a systematic literature review on prospective studies including patients with previous syncope and inducible VA (Supplementary Table S5). The initial literature search resulted in 73 studies, of which 17 were found to be prospective and therefore eligible for systematic analysis. In summary, besides one registry investigating the context in which PVS is used in ICD recipients, that also included patients with previous syncope and inducible ventricular tachycardia but did not focus on these patients in detail^9^, our here presented study is—to the best of our knowledge—the largest prospective one analyzing patients with previous syncope and inducible VA. Other prospective reports focused on methods including an abnormal signal-averaged electrocardiogram to predict VA inducibility and assessed the value of PVS in different subgroups of patients with syncope. Our conducted review of classes of recommendations and levels of evidence for ICD implantation in this patient population according to international guidelines^2,7,8^ further underlines that previously available evidence is heterogeneous, partly controversial, and data are still somehow limited to mainly single-center and retrospective studies (Supplementary Table S6).

## Discussion

**Figure 3:**
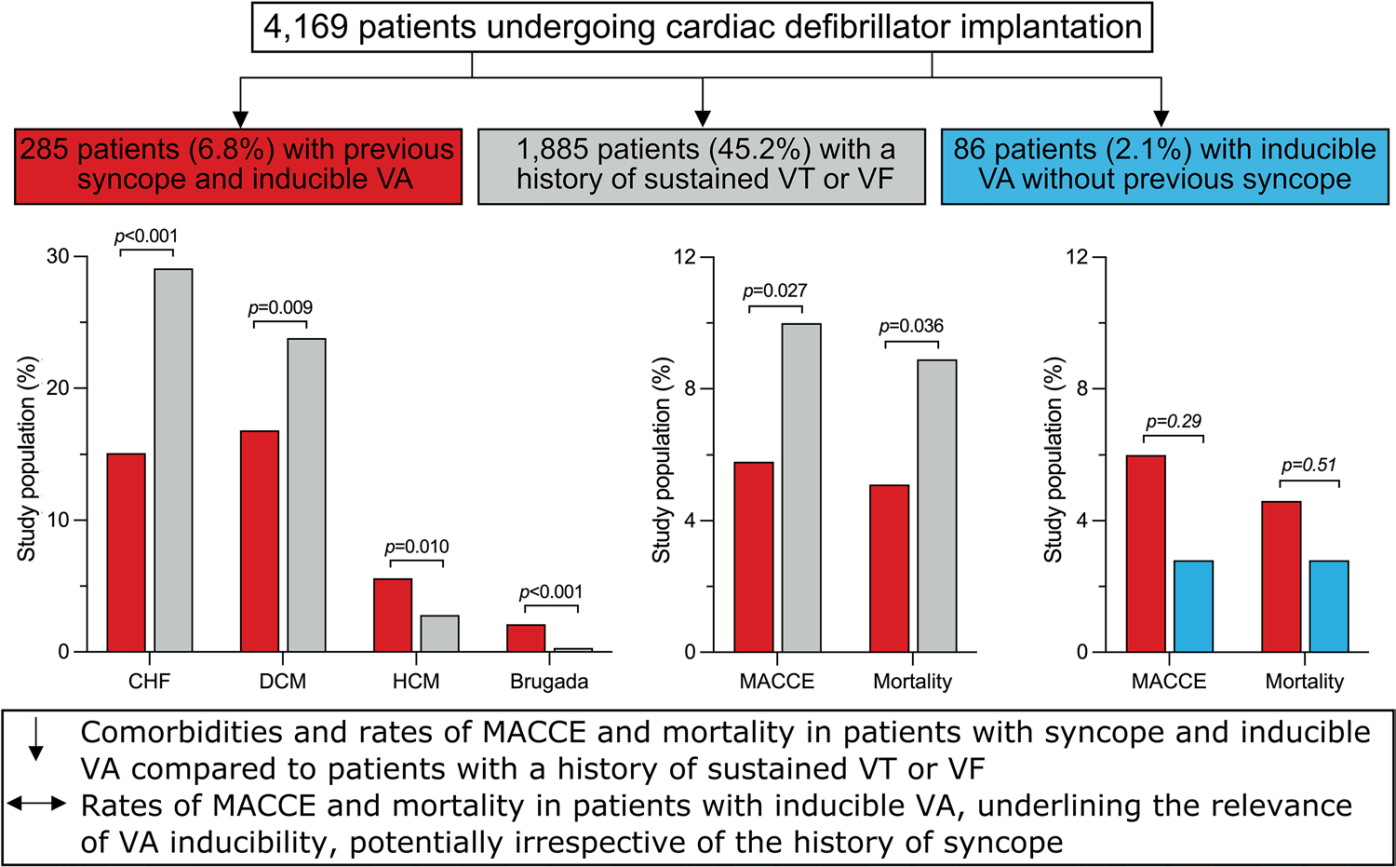
Central illustration. Patients with syncope and inducible VA undergoing defibrillator implantation present less often with most cardiac comorbidities and have lower rates of 1-year-mortality and MACCE than patients with a history of sustained VT or VF. Rates of 1-year-mortality and MACCE are comparable in patients with inducible VA with versus without syncope. *CHF* congestive heart failure, *DCM* dilated cardiomyopathy, *HCM* hypertrophic cardiomyopathy, *MACCE* major adverse cardiac or cerebrovascular event, *VA* ventricular arrhythmia, *VF* ventricular fibrillation, *VT* ventricular tachycardia.

The main findings of the present study are as follows: Patients with syncope and inducible VA undergoing implantation of an ICD with or without CRT, who only represent a small part of all patients undergoing device implantation, (1) present less often with most cardiac comorbidities and (2) have lower rates of 1-year-mortality and MACCE than patients with a secondary preventive indication, and (3) have similar rates of post-procedural adverse events compared to patients undergoing defibrillator implantation for inducible VA without previous syncope (Fig. 3).

### Patient characteristics and post-procedural adverse events

Multiple cardiovascular risk factors may be associated with the occurrence of syncope in the general population and more specifically, in patients with congestive heart failure^10–12^. Especially patients with recurrent syncope have been found to be more likely to present with coronary artery disease, prior diagnosis of arrhythmia and electrocardiogram abnormalities^13^. However, pathophysiology as well as related clinical features vary among different populations and may not be consistently based on a cardiovascular risk profile making evaluation of patients with syncope challenging^5,14^. In the here presented study reporting data from a prospective, multi-center registry, patients with a history of syncope presented less often with any structural heart disease than those with a secondary preventive indication suggesting that heterogeneous rather than solely cardiac reasons contribute to the development of syncope. Still, patients with syncope more frequently presented with hypertrophic cardiomyopathy and Brugada syndrome, which are both known to predispose the occurrence of syncope resulting in a prevalence of up to 30%^15,16^.

The prognosis of a syncope differs depending on its etiology, with 1-year-mortality ranging from 0% for vasovagal events up to 30% in the presence of heart disease^17^. In patients undergoing ICD implantation for primary prevention of SCD, those with a history of syncope have been reported to be at higher risk for VA, appropriate shocks and antitachycardia pacing therapies compared to those without previous syncope^17^. Our findings now help to clarify that patients undergoing defibrillator implantation for a history of syncope and inducible VA have lower rates of 1-year-mortality and MACCE than patients with a secondary preventive indication. This might be mainly explained by fewer cardiac comorbidities as the number of serious adverse events in patients undergoing secondary preventive defibrillator implantation has been reported to vary depending on the underlying cardiac diagnosis with patients with ischemic or dilated cardiomyopathy, which are both present in the majority of the here enrolled patients with a secondary preventive indication, being at higher risk for ICD therapies than those with hypertrophic cardiomyopathy or channelopathies^18,19^. However, similar rates of ICD shocks, rehospitalizations and electrical storm during follow-up support the indication for defibrillator implantation in patients with syncope and inducible VA.

As opposed to a previous report^20^, the recurrence rate of syncope was low, whereas patients had a relatively high probability of receiving ICD shocks. This might be due to rapid delivery of effective therapies resulting in prevention of recurrent syncope. Whereas in recent years, long detection times are usually recommended to decrease the number of ICD therapies^21,22^, in selected patients, e.g., those at high risk for syncope, it might be reasonable to rather use conventional programming. Another reason for the low recurrence rate might be that it has been found to increase with the number of previously experienced episodes^14^. Antitachycardia pacing might have further not only affected the number of experienced syncopes, but also patient-reported outcomes during follow-up. Consequently, one might speculate that different patient and defibrillator programming characteristics might have led to fewer post-procedural adverse events in patients with syncope than previously described^11,17–19^. However, this was beyond the scope of the present study and the incidence of appropriate ICD shocks still needs to be determined in this cohort of patients.

### Importance of ventricular arrhythmia inducibility

Inducible VA have been shown to identify patients with coronary artery disease at increased risk for overall and arrhythmia-related death, especially if left ventricular ejection fraction is ≥ 30%^23^. In contrast, PVS might play a minor role for non-ischemic cardiomyopathy due to low inducibility, low reproducibility and low predictive value of induced VA^5^ underlining that the recommendations for the indication of PVS are based on limited evidence as it still holds true^2^.

Among patients introducing with syncope, the presence of structural heart disease with reduced left ventricular ejection fraction or VA inducibility during PVS is highly suggestive that syncope is related to VA and that ICD implantation can be useful in these patients regardless of the timing of past revascularization^5^. Our findings including patients from a prospective, multi-center database now emphasize the relevance of VA inducibility, irrespective of the history of syncope. Despite less frequently found ischemic cardiomyopathy in patients with than without syncope and inducible VA, ICD shocks (16.0% vs. 15.0%) and rehospitalization (27.6% vs. 21.7%) were present in a comparable number of patients during follow-up, without any differences in the rate of 1-year-mortality and MACCE between both groups. Low rates of SCD and recurrent syncope are concomitant with a high positive predictive value supporting the use of defibrillators in patients with inducible VA^5^. Consequently, patients with inducible VA, who are not well represented in trials so far but may be at risk to be underdiagnosed due to fewer cardiac comorbidities, experience a relevant number of post-procedural adverse events not differing between those with vs. without syncope, which supports the indication for defibrillator implantation in this population. Besides one registry investigating the context in which PVS is used in ICD recipients, that also included patients with previous syncope and inducible ventricular tachycardia but did not focus on these patients in detail^9^, our here presented study is—to the best of our knowledge—the largest prospective one reporting demographics, data on device implantation and post-procedural adverse events during the first time period after ICD implantation in this patient population. As it is further one of only a few multi-center studies and consists of a large group of patients with different underlying structural heart diseases, our study enables insights into a more representative patient sample, increases generalizability of findings and helps to put this relatively rare patient population in relation to a well-studied control group. Importantly, so far, literature and guidelines are heterogeneous, partly controversial^2,7,8^, and data are still somehow limited to mainly single-center and retrospective studies consisting of only relatively small numbers of patients with syncope and inducible VA (Supplementary Tables S5 and S6)^24^. Since decision making is relevant in clinical practice, our here presented data appear ethically warranted as a piece of a challenging and incomplete puzzle contributing to a better understanding of this rarely described population.

### Study limitations

The present study has some limitations. In a registry-based cohort, potential confounding factors and a reporting bias have to be considered, as follow-up was only centralized at 1 year post-implantation and performed during clinical routine before. Device implantations and post-procedure care were left to each participating center and are therefore heterogeneous in nature. The follow-up was limited to 1 year after ICD implantation. However, our study aimed to concentrate on post-procedural events during the first time period after ICD implantation, which has only been rarely described, paving the way for subsequent trials including a long-term follow-up. Whether our here presented findings are transferable to a long-term follow-up remains speculative but is supported by the landmark MUSTT data^3^, reporting that non-inducible patients have a significantly lower risk of cardiac arrest or sudden death as well as mortality compared with inducible patients both in the short- and long-term. This observation might underline the value of the here presented differences in post-procedural endpoints that are already identifiable after 1 year of follow-up in patients with previous syncope and inducible VA. Considering the investigated patients, e.g., those after myocardial infarction with reduced left ventricular ejection fraction and unexplained syncope after non-invasive evaluation, who have inducible VA during PVS, a potential overlap with primary prevention ICD indication cannot be excluded. Another limitation is that patient inclusion was between 2007 and 2010 which may challenge interpretation concerning today’s treatment. Heart failure medication has changed since then and programming of ICDs has become much less aggressive (higher VA detection rates, longer detection duration). However, the here investigated study cohort corresponds to the currently available guidelines still representing a small part of all patients undergoing ICD implantation, which underlines the clinical relevance of the presented data even after development of heart failure medication and ICD programming. In the future with the updated ESC guideline recommendation, patients not being inducible during PVS might serve as a valuable control group but have not been included in the here investigated registry. Nonetheless, the German Device Registry enables insights into a large, prospective real-world cohort undergoing defibrillator implantation for syncope and inducible VA, which has not been described before.

## Conclusion

Patients with syncope and inducible VA undergoing defibrillator implantation, who only represent a small part of all patients undergoing device implantation, present less often with most cardiac comorbidities and have lower rates of 1-year-mortality and MACCE than patients with a secondary preventive indication. Numbers of post-procedural adverse events are comparable in patients with inducible VA with vs. without syncope, which underlines the relevance of VA inducibility, potentially irrespective of the history of syncope.

## Supplementary Information

Supplementary Tables.

## Data Availability

The underlying data are available from the corresponding author upon reasonable request.
