# Supplementary material for: Cardiac defibrillator implantation in patients with syncope and inducible ventricular arrhythmia: insights from the German Device Registry
Source: Sci Rep. 2023 Jul 27;13:12182. doi: 10.1038/s41598-023-37440-2 (PMC10374635; doi:10.1038/s41598-023-37440-2)
Supplement: Supplementary file 1 — Supplementary Tables. [file 41598_2023_37440_MOESM1_ESM.pdf]

Supplemental Material

Cardiac defibrillator implantation in patients with syncope and inducible ventricular arrhythmia: insights from the German Device Registry

Ann-Kathrin Kahle, MD, Jochen Senges, MD, Matthias Hochadel, PhD, Johannes Brachmann, MD, Dierk Thomas, MD, Florian Straube, MD, Klaus Bonaventura, MD, Robert Larbig, MD, Nikos Werner, MD, Christian Butter, MD, Fares-Alexander Alken, MD, Christian Meyer, MD, MA

Supplementary Table S1. Baseline characteristics of patients undergoing first-time defibrillator implantation.

Variable	Syncope and inducible VA (n=208)	Secondary preventive indication (n=1,309)	*p* value	Odds ratio (95%-CI)
Age, years	65.2±15.0	64.3±14.0	0.18	---
Male sex	170 (81.7)	1,041 (79.5)	0.46	1.15 (0.79-1.68)
Body mass index, kg/m^2	25.0 (23.5–29.7)	26.1 (24.2–29.4)	0.60	---
LVEF, %	43.6±14.1	38.7±14.5	<0.001	---
LVEF ≤30%	47 (24.6)	495 (40.2)	<0.001	0.49 (0.34-0.69)
Sinus rhythm at baseline	174 (83.7)	1,036 (79.4)	0.15	1.33 (0.90-1.97)
Atrial fibrillation at baseline	29 (13.9)	228 (17.5)	0.21	0.77 (0.50-1.16)
Atrioventricular block	45 (21.6)	192 (14.7)	0.011	1.60 (1.11-2.30)
Atrioventricular block III	0 (0)	23 (6.8)	0.16	---
Intraventricular conduction disorder	61 (29.3)	414 (31.8)	0.48	0.89 (0.65-1.23)
LBBB	35 (16.8)	250 (19.2)	0.42	0.85 (0.58-1.26)
RBBB	9 (4.3)	99 (7.6)	0.089	0.55 (0.27-1.11)
Structural heart disease	179 (86.1)	1,182 (90.3)	0.061	0.66 (0.43-1.02)
Coronary artery disease	120 (57.7)	814 (62.2)	0.22	0.83 (0.62-1.12)
Prior myocardial infarction	70 (33.7)	452 (34.5)	0.80	0.96 (0.71-1.31)
Time since myocardial infarction, months	137 (101–203)	45 (0–139)	0.004	---
Coronary artery bypass graft	27 (13.0)	209 (16.0)	0.27	0.79 (0.51-1.21)
Dilated cardiomyopathy	33 (15.9)	326 (24.9)	0.004	0.57 (0.38-0.84)
Hypertrophic cardiomyopathy	9 (4.3)	39 (3.0)	0.30	1.47 (0.70-3.08)
Congestive heart failure	32 (15.4)	389 (29.7)	<0.001	0.43 (0.29-0.64)
Time since diagnosis, months	4 (0–30)	3 (0–20)	1	---
Hypertensive heart disease	17 (8.2)	68 (5.2)	0.083	1.62 (0.93-2.82)
Primary electrical disease	13 (6.3)	48 (3.7)	0.079	1.75 (0.93-3.29)
Brugada syndrome	6 (2.9)	4 (0.3)	<0.001	9.68 (2.71-34.61)
Long QT	2 (1.0)	27 (2.1)	0.28	0.46 (0.11-1.95)
ARVC	1 (0.5)	9 (0.7)	0.73	0.70 (0.09-5.53)
Arterial hypertension	115 (55.3)	621 (47.4)	0.035	1.37 (1.02-1.84)
Diabetes mellitus	34 (16.3)	306 (23.4)	0.024	0.64 (0.43-0.95)
Stroke	6 (2.9)	51 (3.9)	0.48	0.73 (0.31-1.73)
Chronic obstructive pulmonary disease	2 (1.0)	43 (3.3)	0.067	0.29 (0.07-1.19)
Chronic kidney disease	24 (11.5)	184 (14.1)	0.33	0.80 (0.51-1.25)

Values are presented as mean ± SD, median (IQR) or n (%). Variables were compared using the Mann-Whitney *U* test or the chi-square test, respectively. ARVC, arrhythmogenic right ventricular cardiomyopathy; CI, confidence interval; LBBB, left bundle branch block; LVEF, left ventricular ejection fraction; RBBB, right bundle branch block; VA, ventricular arrhythmia.

Supplementary Table S2. Post-procedural adverse events during 1 year of follow-up of patients undergoing first-time defibrillator implantation.

Variable	Syncope and inducible VA (n=208)	Secondary preventive indication (n=1,309)	*p* value	Odds/Hazard ratio (95%-CI)
1-year-mortality[a]	4.0	8.7	0.023	0.45 (0.22-0.91)
Survival time, months	10.8 (5.0–21.2)	7.9 (3.1–15.5)	0.32	---
MACCE	4.4	9.4	0.019	0.45 (0.22-0.89)
Cardiopulmonary resuscitation	0	1.0	0.20	---
Syncope	6.1	5.6	0.83	1.10 (0.48-2.53)
ICD shock	20.6	24.0	0.34	0.82 (0.55-1.23)
VT storm / incessant VT	3.6	2.5	0.42	1.45 (0.58-3.59)
Ablation procedure	7.9	4.3	0.16	1.91 (0.77-4.76)
Myocardial infarction	0.7	1.2	0.55	0.54 (0.07-4.22)
Stroke	0	0.8	0.28	---
Revascularization	1.0	3.2	0.22	0.30 (0.04-2.30)
Device revision	4.8	7.0	0.28	0.66 (0.31-1.41)
Rehospitalization	35.2	38.5	0.42	0.87 (0.61-1.23)
For device-related reasons	16.7	12.9	0.20	1.35 (0.86-2.13)
For other cardiac reasons	7.4	12.1	0.085	0.58 (0.31-1.08)
Number of hospitalizations	1 (1–2)	1 (1–2)	0.094	---
Duration of hospitalization, days	10 (5–20)	10 (5–20)	0.79	---

Values are presented as % or median (IQR). Variables were compared using the chi-square or the Mann-Whitney *U* test.
[a] Kaplan-Meier estimates at 366 days after index discharge, compared by log-rank test (hazard ratio).
CI, confidence interval; ICD, implantable cardioverter-defibrillator; MACCE, major adverse cardiac or cerebrovascular event; VA, ventricular arrhythmia; VT, ventricular tachycardia.

Supplementary Table S3. Baseline characteristics of patients with inducible VA undergoing first-time defibrillator implantation.

Variable	Syncope and inducible VA (n=180)	Inducible VA without syncope (n=53)	*p* value	Odds ratio (95%-CI)
Age, years	65.5±15.3	64.7±12.6	0.37	---
Male sex	148 (82.2)	45 (84.9)	0.65	0.82 (0.35-1.91)
LVEF, %	43.7±14.4	43.0±15.5	0.70	---
Sinus rhythm at baseline	147 (81.7)	50 (94.3)	0.025	0.27 (0.08-0.91)
Atrial fibrillation at baseline	28 (15.6)	3 (5.7)	0.062	3.07 (0.89-10.53)
Atrioventricular block	42 (23.3)	8 (15.1)	0.20	1.71 (0.75-3.92)
Intraventricular conduction disorder	51 (28.3)	12 (22.6)	0.41	1.35 (0.66-2.78)
LBBB	31 (17.2)	6 (11.3)	0.30	1.63 (0.64-4.15)
RBBB	6 (3.3)	2 (3.8)	0.88	0.88 (0.17-4.49)
Coronary artery disease	105 (58.3)	36 (67.9)	0.21	0.66 (0.35-1.26)
Prior myocardial infarction	60 (33.3)	24 (45.3)	0.11	0.60 (0.32-1.13)
Coronary artery bypass graft	21 (11.7)	9 (17.0)	0.31	0.65 (0.28-1.51)
Cardiomyopathy	35 (19.4)	5 (9.4)	0.089	2.32 (0.86-6.25)
Dilated cardiomyopathy	26 (14.4)	5 (9.4)	0.35	1.62 (0.59-4.45)
Hypertrophic cardiomyopathy	9 (5.0)	0 (0)	0.097	---
Hypertensive heart disease	16 (8.9)	3 (5.7)	0.45	1.63 (0.46-5.81)
Primary electrical disease	12 (6.7)	3 (5.7)	0.79	1.19 (0.32-4.39)
Brugada syndrome	6 (3.3)	0 (0)	0.18	---
Long QT	2 (1.1)	0 (0)	0.44	---
ARVC	0 (0)	2 (3.8)	0.009	---
Arterial hypertension	104 (57.8)	30 (56.6)	0.88	1.05 (0.57-1.95)
Diabetes mellitus	32 (17.8)	9 (17.0)	0.89	1.06 (0.47-2.38)
Stroke	5 (2.8)	1 (1.9)	0.72	1.49 (0.17-13.00)
Chronic obstructive pulmonary disease	1 (0.6)	1 (1.9)	0.36	0.29 (0.02-4.72)
Chronic kidney disease	24 (13.3)	5 (9.4)	0.45	1.48 (0.53-4.08)

Values are presented as mean ± SD, median (IQR) or n (%). Variables were compared using the Mann-Whitney *U* test or the chi-square test, respectively. ARVC, arrhythmogenic right ventricular cardiomyopathy; CI, confidence interval; LBBB, left bundle branch block; LVEF, left ventricular ejection fraction; RBBB, right bundle branch block; VA, ventricular arrhythmia.

Supplementary Table S4. Post-procedural adverse events during 1 year of follow-up of patients with inducible VA undergoing first-time defibrillator implantation.

Variable	Syncope and inducible VA (n=180)	Inducible VA without syncope (n=53)	*p* value	Odds/Hazard ratio (95%-CI)
1-year-mortality[a]	4.6	0	0.12	---
MACCE	5.1	1.9	0.31	2.82 (0.35-22.78)
Syncope	4.5	0	0.29	---
ICD shock	15.8	16.7	0.89	0.94 (0.39-2.28)
VT storm / incessant VT	2.1	0	0.31	---
Rehospitalization	26.8	31.3	0.56	0.81 (0.39-1.65)
Improvement in quality of life	47.5	53.2	0.50	0.80 (0.41-1.54)
Feeling of safety during treatment	98.0	81.8	0.026	10.67 (0.87-130.44)

Values are presented as % or median (IQR). Variables were compared using the chi-square or the Mann-Whitney *U* test.
[a] Kaplan-Meier estimates at 366 days after index discharge, compared by log-rank test (hazard ratio).
CI, confidence interval; ICD, implantable cardioverter-defibrillator; MACCE, major adverse cardiac or cerebrovascular event; VA, ventricular arrhythmia; VT, ventricular tachycardia.

Supplementary Table S5. Systematic literature review of prospective studies on patients with previous syncope and inducible VA.

Reference, year of publication	Aim of the study, study type, number of patients, enrolment period	Main results	Conclusions
Morady et al., J Am Coll Cardiol., 1983	Aim of the study: To clinically follow-up pts. with recurrent unexplained syncope who had been evaluated by PVS.<br>Study type: Single-center<br>Number of pts.: 53<br>Enrolment period: NA	• NSVT was induced in 15 (28%), SMVT in 9 (17%) and VF in 4 (8%), sinus node function was abnormal 2 (4%).<br>• Female sex and lack of structural heart disease were independently associated with negative PVS ($p<0.001$).<br>• The recurrence rate of syncope was 43% during a follow-up of 31±10 months in pts. with negative PVS, 40% during 22±6 months in pts. with inducible NSVT, 0% during 30±12 months in pts. with inducible SMVT and 25% during 21±10 months in pts. with inducible VF.	In pts. with recurrent unexplained syncope undergoing PVS, a potential cause of syncope is least likely to be found in women without structural heart disease. Whereas the finding of SMVT is likely to be clinically significant, with no recurrence of syncope during AAD therapy, polymorphic NSVT or VF may be nonspecific, unrelated to syncope.
Teichman et al., Am Heart J., 1985	Aim of the study: To examine the diagnostic yield and therapeutic efficacy of EPS in pts. with unexplained syncope.<br>Study type: Single-center<br>Number of pts.: 150<br>Enrolment period: NA	• VA were induced in 36 (22%) pts.<br>• During a mean follow-up of 31 months, syncope recurred in 47% without and in 15% of pts. with abnormal EPS findings despite initiated therapy ($p<0.0005$).	EPS is useful in assessing the causes of unexplained syncope and in directing therapy, and a relevant number of pts. benefit from EPS during follow-up.
Lindsay et al., Circulation, 1986	Aim of the study: To determine if frequency analysis facilitates identification of pts. who will have sustained VT induced during PVS.<br>Study type: Single-center<br>Number of pts.: 58 (group 1 = 20 with documented sustained VT or cardiac arrest, prospective group 2 = 12 with NSVT + 26 with syncope)<br>Enrolment period: NA	• In group I, SMVT was induced in 18 pts., each with an area ratio value >20, SMVT was not induced in 2 pts., each with an area ratio value <20.<br>• SMVT was induced in 5/12 pts. in group II with abnormal FFT values and in 0/26 pts. with normal FFT values.<br>• Multivariate analysis demonstrated that area ratio values were independent of other determinants of inducibility, including LVEF and prior myocardial infarction.	FFT values may improve identification of pts. in whom SMVT can be induced during PVS.
Winters et al., J Am Coll Cardiol., 1987	Aim of the study: To analyze whether the presence of late potentials recorded from signal averaging of the surface QRS complex correlates with VT inducibility in pts. with unexplained syncope.<br>Study type: Single-center<br>Number of pts.: 40 (6 with and 34 without BBB)<br>Enrolment period: NA	• Of 34 pts. without BBB, 12 had inducible VT (group I) and 22 had not (group II).<br>• ≥1 abnormal signal averaging variables were present in 92% vs. 27% of pts. in group I vs. II ($p<0.005$), with an abnormal root mean square voltage of the terminal 40 ms being the most significant distinguishing variable and present in 83% vs. 14% of pts. ($p<0.005$). The QRS vector duration was prolonged in 58% vs. 9% ($p<0.05$), the duration of low amplitude signals in 58% vs. 19% ($p<0.05$).<br>• The presence of abnormal signal	Signal averaging of the surface QRS complex is a useful noninvasive technique for selecting pts. with unexplained syncope who should undergo PVS.

		averaging variables was more predictive of inducible VT than 24 hour ambulatory ECG monitoring.<br>• An abnormally low root mean square voltage of the terminal 40 ms had the highest sensitivity (82%) and specificity (91%) in distinguishing pts. with unexplained syncope who had inducible VT.	
Nalos et al., J Am Coll Cardiol., 1987	Aim of the study: To assess the clinical utility of the signal-averaged ECG in a large group of high-risk pts. referred for PVS.<br>Study type: Single-center<br>Number of pts.: 100 (38 with syncope, 24 with NSVT, 25 with SMVT, 13 with sudden cardiac arrest)<br>Enrolment period: 1985-1986	• SMVT was induced in 29% of pts.<br>• Pts. with vs. without inducible VT differed regarding abnormal signal-averaged ECGs ($p \leq 0.001$).<br>• The sensitivity and specificity of signal averaging for predicting the induction of SMVT were 93% and 94%. The signal-averaged ECG was the best predictor of induction of SMVT, independent of LVEF, presence of ventricular aneurysm, myocardial infarction and other clinical variables ($p<0.0001$).	The signal-averaged ECG is a sensitive and specific predictor for the induction of SMVT.
Kaul et al., PACE, 1988	Aim of the study: To assess the role of EPS, including PVS and ajmaline stress, in determining the etiology of unexplained syncope in pts. with BBB.<br>Study type: Single-center<br>Number of pts.: 35 (20 with and 15 without structural heart disease)<br>Enrolment period: NA	• SMVT was inducible in 9 (25.7%) and polymorphic VT in 2 pts. (5.7%). LVEF was <40% in 5 pts. (45.5%) with inducible VT. 2 pts. had a co-existence of inducible HV block and VT.<br>• The incidence of inducible VT was higher (45% vs. 13.3%), and the presence of negative studies was non-significantly lower (30% vs. 53.3%) in pts. with structural heart disease compared to those without.<br>• During a mean follow-up of 16.5±9.2 months, pts. with inducible SMVT (except one with poor LVEF who died suddenly) have been asymptomatic on AAD.	VT accounts for syncope in a significant number of pts., long-term management guided by PVS is successful in preventing recurrent syncope in pts. with inducible arrhythmias such as SMVT.
Kuck et al., Eur Heart J., 1988	Aim of the study: To evaluate ventricular vulnerability in different subgroups of pts. with hypertrophic cardiomyopathy.<br>Study type: Single-center<br>Number of pts.: 54 (3 with a history of cardiac arrest due to VT (group A), 8 with a history of unexplained syncope (group B), 43 were 'asymptomatic' (group C)).<br>Enrolment period: NA	• VA were induced in 18 pts., including repetitive ventricular response in 6 pts., NSVT in 4, SMVT in 5, and VF in 3. VT was polymorphic in 6 pts. and monomorphic in 3.<br>• There were no differences in the type and incidence of VA between groups (group A: 67% vs. group B: 38% vs. group C: 30%). Disregarding induced ventricular responses, VA were induced in 36% of symptomatic vs. 19% of asymptomatic pts.	The type and incidence of induced VA does not differ between symptomatic vs. asymptomatic pts. with hypertrophic cardiomyopathy.
Turitto et al., Am J Cardiol., 1988	Aim of the study: To assess the predictive accuracy of different clinical variables for the induction of SMVT.<br>Study type: Single-center<br>Number of pts.: 105 (22 with	• LVEF <40%, history of syncope or presyncope and abnormal signal-averaged ECG were more common in pts. with inducible VT than in noninducible pts., there were no differences between pts. with inducible VF and noninducible pts.<br>• The sensitivity, specificity and	The history of syncope does not generally predict induction of sustained VT/VF. Abnormal signal-averaged ECG is the most accurate predictor for VT/VF induction.

	induced SMVT, 14 with induced VF, 69 pts. without induced SMVT/VF)<br><br><u>Enrolment period:</u> NA	predictive accuracy of the signal-averaged ECG for the induction of SMVT were 64%, 89% and 84%.<br>• The signal-averaged ECG was found to be the single most accurate screening test to predict the inducibility of SMVT in pts. with NSVT, independent of the etiology of heart disease and the length of spontaneous runs.	
Manolis et al., Am J Cardiol., 1990	<u>Aim of the study:</u> To examine the value of PVS in assessing the inducibility of VA, guiding therapy and predicting the clinical outcome in pts. with spontaneous NSVT.<br><br><u>Study type:</u> Single-center<br><br><u>Number of pts.:</u> 52 with NSVT (5 with palpitations, 11 with presyncope, 26 with syncope, 10 were asymptomatic)<br><br><u>Enrolment period:</u> 1983-1989	• Arrhythmia was inducible in 21 pts. (40%), who all received AAD during follow-up.<br>• The incidence of syncope was lower in pts. with vs. without inducible arrhythmia ($p<0.05$).<br>• During a follow-up of 21±17 months, there was no SMVT in either group, and 6 (29%) vs. 3 pts. (10%) died (p=NS).	Syncope does not necessarily predict inducible VA. AAD is efficient to prevent arrhythmias in pts. with inducible VT.
Brembilla-Perrot et al., Am Heart J., 1991	<u>Aim of the study:</u> To assess the response to PVS and the clinical outcome in pts. with idiopathic dilated cardiomyopathy.<br><br><u>Study type:</u> Single-center<br><br><u>Number of pts.:</u> 103 (11 with spontaneous VT including 3 with explained syncope, 92 without documented SMVT including 13 with syncope).<br><br><u>Enrolment period:</u> NA	• SMVT was induced in 8/11 pts. with spontaneous sustained VT, in 0/36 without VA during Holter monitoring, and in 7/56 with VPBs, of whom 5 had syncope.<br>• During follow-up, there were 8 sudden deaths among pts. who initially had syncope, inducible sustained VT, or both and 3 episodes of SMVT in pts. who initially had NSVT but inducible SMVT.	PVS may be indicated in patients with initial syncope and sustained VT.
Steinberg et al., J Am Coll Cardiol., 1994	<u>Aim of the study:</u> To determine the predictors of inducible VT in pts. with unexplained syncope and to examine the value of the signal-averaged ECG.<br><br><u>Study type:</u> Multi-center (n=6)<br><br><u>Number of pts.:</u> 168<br><br><u>Enrolment period:</u> 1998-1991	• VT was induced in 28 pts. (15%), predicted by previous myocardial infarction, reduced LVEF and abnormal signal-averaged ECG results.<br>• The signal-averaged ECG was the most sensitive test, but with poor specificity.<br>• Pts. with both previous myocardial infarction and an abnormal signal-averaged ECG had a 17-fold increased risk of inducible VT.	The signal-averaged ECG is the most sensitive noninvasive predictor for inducible VT, but false positive in many pts., a combination with the history of previous myocardial infarction is the most efficient screening method.
Englund et al., J Am Coll Cardiol., 1995	<u>Aim of the study:</u> To examine the inducibility of VA in pts. with bifascicular block both with and without a history of syncope.<br><br><u>Study type:</u> Single-center<br><br><u>Number of pts.:</u> 101 (41 with unexplained syncope and 60 were asymptomatic)	• PVS resulted in sustained VA in 18 pts. (18%), 8 in the syncope and 10 in the asymptomatic group (p=NS), including 3 in each group with SMVT.<br>• During a mean follow-up of 21 months, 4 pts. experienced sudden death, 5 syncope and 1 appropriate ICD discharges. One of these pts. had an inducible VA at baseline.	Inducibility of VA in pts. with bifascicular block does not differ between those with vs. without syncope. Inducible VA does not predict clinical events during follow-up.

	Enrolment period: 1991-1993		
Olshansky et al., Am Heart J., 1999	Aim of the study: To compare mortality rates of pts. enrolled with syncope to those enrolled with spontaneous VA.<br><br>Study type: Multi-center, randomized (n=13)<br><br>Number of pts.: 486 (25 with syncope only, 24 with syncope and VT, 332 with VT only, 105 with VF)<br><br>Enrolment period: 1985-1991	• At 1 and 4 years, arrhythmic and total mortality rates did not differ. At 4 years, arrhythmic death in pts. with syncope alone was 37%, in those with syncope and VT 41%, in those with VT alone 31%, and in pts. with VF 39%.<br>• 81 pts. (16.7%) underwent ICD implantation, with the greatest percentage in the syncope-VT group (42% vs. 12% of the pts. with syncope alone vs. 14% of the pts. with VT alone vs. 19% of the pts. with VF, p=0.005).	Syncope associated with induced VT indicates high risk for death, similar to that of pts. with spontaneous VT.
Brembilla-Perrot et al., Europace, 2001	Aim of the study: To investigate the diagnostic value of different techniques for the evaluation of the mechanism of syncope in pts. with myocardial infarction and BBB and their prognosis.<br><br>Study type: Single-center<br><br>Number of pts.: 130 (81 with RBBB, 49 with LBBB)<br><br>Enrolment period: 1989-1999	• 68% of pts. had inducible VT.<br>• The sensitivity and specificity of NSVT on Holter monitoring for VT detection were 42.5% and 47% in pts. with RBBB, 62% and 36% in those with LBBB; sensitivity and specificity of LVEF <40% were 67.5% and 65% in pts. with RBBB, 85% and 9% in those with LBBB; sensitivity and specificity of the combination of 2 of the 3 signal-averaged ECG criteria (QRS duration >155 ms, LAS duration >30 ms and RMS 40 <17 µV) were 50% and 57% in pts. with RBBB; sensitivity and specificity of the combination of 2 of the 3 criteria (QRS duration >165 ms, LAS duration >40 ms and RMS 40 <17 µV) were 73% and 55.5% in pts. with LBBB.<br>• During a follow-up of 4.7±2.5 years, 12 pts. died suddenly and 12 died from heart failure. In univariate and multivariate analysis, only VT induction was a predictor of sudden death, a long QRS duration (>165 ms) and VT induction independently predicted total cardiac mortality.	Sudden death is only predicted by the induction of SMVT. Considering the high incidence of inducible SMVT, the low value of Holter monitoring and decreased LVEF for the prediction of VA and the poor prognosis of pts. with inducible VT and low LVEF, systematic PVS is indicated in pts. with myocardial infarction, syncope and BBB, irrespective of the non-invasive studies results.
Steinberg et al., J Cardiovasc Electrophysiol., 2001	Aim of the study: To clarify the prognosis and recurrent event rate, clinical course, and risk factors for outcome events, and to assess the apparent impact of device and drug therapy in pts. with syncope and inducible VT.<br><br>Study type: Multi-center (n=13) substudy of the randomized AVID trial<br><br>Number of pts.: 80 in the substudy and 429 from the AVID registry with "out of hospital syncope with structural heart disease and inducible VT/VF with symptoms"	• Of the substudy pts., 21 (26%) had inducible polymorphic VT/VF, 11 (14%) had SMVT <200 beats/min, and 48 (60%) had SMVT ≥200 beats/min.<br>• The ICD was used as sole therapy in 75% of pts. (and with AAD in an additional 9%) and in 59% of the syncope registry pts.<br>• Survival rates at 1 and 3 years were 93% and 74% for the substudy pts. and 90% and 74% for the registry pts.<br>• Survival of the substudy pts. was similar to the VT pts. treated by ICD and superior to the VT pts. treated by AAD (p=0.05) in the randomized main trial.<br>• Mortality events in the substudy were	The ICD should be used as primary antiarrhythmic therapy in pts. with unexplained syncope, structural heart disease, and inducible VT/VF during PVS.

	Enrolment period: 1993-1997	marginally predicted by LVEF (p=0.06) but not by inducible VT. The significant predictor of increased mortality in the registry was age (p=0.003) and of reduced mortality was treatment with ICD (p=0.006).	
Brodsky et al., Am Heart J., 2002	Aim of the study: To determine the context in which EPS is used in pts. receiving an ICD.<br>Study type: Multi-center (n=56) AVID study.<br>Number of pts.: 572<br>Enrolment period: 1993-1997	• 384 (67%) pts. had inducible SMVT or VF.<br>• Inducible patients were more likely to have coronary artery disease, previous infarction, and VT as their index arrhythmic event.<br>• The index event of syncope and VT was 26% vs. 18% for inducible vs. noninducible pts., the incidence of history of syncope 13% vs. 14%.<br>• Inducibility of VT or VF did not predict death or recurrent VT or VF.	Syncope does not predict inducible VT/VF. Results from EPS, especially VF induction, should be interpreted with caution.
Cheng et al., PACE, 2012	Aim of the study: To determine the value of EPS in pts. with VF, VT with syncope, or SMVT in the setting of left ventricular dysfunction.<br>Study type: Multi-center<br>Number of pts.: 33,786 undergoing EPS<br>Enrolment period: 2006-2009	• VT were induced in 46.1% of primary prevention and 54.2% of secondary prevention ICD recipients.<br>• Those undergoing EPS were more likely to have had a history of syncope (35.6% vs. 17.0%; p<0.0001), family history of sudden death, lack of congestive heart failure, narrower QRS intervals, and higher LVEF.	EPS is more often used in pts. with potentially high-risk factors for sudden death such as syncope and a family history of sudden death. When EPS is performed, inducible SMVT are commonly seen highlighting the value of PVS.

BBB, bundle branch block; ECG, electrocardiogram; EPS, electrophysiological study; FFT, fast-Fourier transforms; ICD, implantable cardioverter-defibrillator; LBBB, left bundle branch block; LVEF, left ventricular ejection fraction; NSVT, non-sustained ventricular tachycardia; PVS, programmed ventricular stimulation; RBBB, right bundle branch block; SMVT, sustained monomorphic ventricular tachycardia; VA, ventricular arrhythmia; VF, ventricular fibrillation; VPB, ventricular premature beats; VT, ventricular tachycardia.

Supplementary Table S6. Classes of recommendations and levels of evidence for ICD implantation in patients with syncope and inducible VA according to international guidelines.

	ESC Guidelines 2022	AHA/ACC/HRS Guidelines 2017	CCS/CHRS Guidelines 2016
**Ischemic cardiomyopathy**			
LVEF ≤40% (ESC: despite ≥3 months of optimal medical treatment) + unexplained syncope or NSVT + inducible SMVT	Class IIa<br>Level of evidence B	Class I<br>Level of evidence B-NR	-
**Dilated cardiomyopathy**			
LVEF >35% + ≥2 risk factors (syncope, LGE on CMR, inducible SMVT, pathogenic mutations in PLN, FLNC, and RBM20 genes)	Class IIa<br>Level of evidence C	-	-
**Congenital heart disease**			
Presumed arrhythmic (ESC) or unexplained (AHA) syncope in repaired moderate or severe complexity (AHA) + either at least moderate ventricular dysfunction or inducible SMVT (ESC) or at least moderate ventricular dysfunction or marked hypertrophy + inducible SMVT (AHA)	Class IIa<br>Level of evidence C	Class IIa<br>Level of evidence B-NR	-
**Structural heart disease**			
Syncope of unknown origin + inducible VT	-	-	Strong Recommendation, Moderate-Quality Evidence

CMR, cardiac magnetic resonance imaging; LGE, late gadolinium enhancement; LVEF, left ventricular ejection fraction; NSVT, non-sustained ventricular tachycardia; SMVT, sustained monomorphic ventricular tachycardia; VT, ventricular tachycardia.
